# Application of site and haplotype-frequency based approaches for detecting selection signatures in cattle

**DOI:** 10.1186/1471-2164-12-318

**Published:** 2011-06-16

**Authors:** Saber Qanbari, Daniel Gianola, Ben Hayes, Flavio Schenkel, Steve Miller, Stephen Moore, Georg Thaller, Henner Simianer

**Affiliations:** 1 Animal Breeding and Genetics Group, Department of Animal Sciences, Georg-August University, 37075 Göttingen, Germany; 2 Department of Animal Sciences and Department of Dairy Science, University of Wisconsin-Madison, Madison, Wisconsin 53706, USA; 3 Animal Genetics and Genomics, Primary Industries Research Victoria, 475 Mickleham Rd, Attwood, VIC 3049, Australia; 4 Centre for Genetic Improvement of Livestock, Department of Animal and Poultry Science, University of Guelph, Guelph, Ontario, N1G 2W1 Canada; 5 Department of Agricultural, Food and Nutritional Science, University of Alberta, Edmonton, AB, Canada; 6 Institute of Animal Breeding and Animal Husbandry, Christian-Albrechts-University, 24098 Kiel, Germany

## Abstract

**Background:**

'Selection signatures' delimit regions of the genome that are, or have been, functionally important and have therefore been under either natural or artificial selection. In this study, two different and complementary methods--integrated Haplotype Homozygosity Score (|iHS|) and population differentiation index (F_ST_)--were applied to identify traces of decades of intensive artificial selection for traits of economic importance in modern cattle.

**Results:**

We scanned the genome of a diverse set of dairy and beef breeds from Germany, Canada and Australia genotyped with a 50 K SNP panel. Across breeds, a total of 109 extreme |iHS| values exceeded the empirical threshold level of 5% with 19, 27, 9, 10 and 17 outliers in Holstein, Brown Swiss, Australian Angus, Hereford and Simmental, respectively. Annotating the regions harboring clustered |iHS| signals revealed a panel of interesting candidate genes like SPATA17, MGAT1, PGRMC2 and ACTC1, COL23A1, MATN2, respectively, in the context of reproduction and muscle formation. In a further step, a new Bayesian F_ST_-based approach was applied with a set of geographically separated populations including Holstein, Brown Swiss, Simmental, North American Angus and Piedmontese for detecting differentiated loci. In total, 127 regions exceeding the 2.5 per cent threshold of the empirical posterior distribution were identified as extremely differentiated. In a substantial number (56 out of 127 cases) the extreme F_ST _values were found to be positioned in poor gene content regions which deviated significantly (p < 0.05) from the expectation assuming a random distribution. However, significant F_ST _values were found in regions of some relevant genes such as SMCP and FGF1.

**Conclusions:**

Overall, 236 regions putatively subject to recent positive selection in the cattle genome were detected. Both |iHS| and F_ST _suggested selection in the vicinity of the Sialic acid binding Ig-like lectin 5 gene on BTA18. This region was recently reported to be a major QTL with strong effects on productive life and fertility traits in Holstein cattle. We conclude that high-resolution genome scans of selection signatures can be used to identify genomic regions contributing to within- and inter-breed phenotypic variation.

## Background

The domestication of cattle (*Bos taurus *and *Bos taurus indicus*) 8,000-10,000 years ago [1] had a significant impact on human civilization. Since that time, a broad range of either natural as well as man made factors (e.g., geography, environment, culture and directional artificial selection) has led to diversity in cattle: Today we know more than 800 cattle breeds across the world. The cattle genome therefore represents a significant opportunity for identifying genetic variation that contributes to phenotypic diversity and for detecting genome response to strong directional selection from both domestication and subsequent artificial selection.

Recently a number of studies with different analytical concepts have been conducted to detect signals of recent positive selection on a genome-wide scale in cattle [[2-6] and [7]]. The methods used are based either on the allele frequency spectrum or on properties of haplotypes segregating in populations. For example, comparing F_ST _values among loci provides an estimate of how much genetic variability exists between, rather than within, populations [8,9]. This statistic assumes that geographically variable selective forces favor different variants in different regions. Hence, between-population allele frequency differences may be more extreme in genome regions harboring such variants. The method can be used to scan patterns of variation over many loci. Akey et al. (2002) [10] suggested using the loci in the tails of the empirical distribution as candidate targets of selection. Another approach to infer evidence of past selection is the "Extended Haplotype Homozygosity" (EHH) test [11] which identifies regions with an unusually long range of haplotype and a high population frequency. Voight et al. (2006) [12] developed the "integrated Haplotype Score" (|iHS|), an extension of EHH, based on the comparison of EHH between derived and ancestor alleles within a population. In this concept, directional selection favoring a new mutation results in a rapid increase in the frequency of the selected allele along with the background haplotype in which the mutation arose. This phenomenon increases linkage disequilibrium (LD) on the chromosomes which harbor the derived (selected) allele, but not the unselected allele, which therefore acts as a "control". Thus, this measure is most sensitive to a rapid increase in the frequency of the derived allele at a selected site, but the derived allele must have existed only on a distinct background (haplotype) prior to selection and must not have reached fixation yet [12,13]. After fixation, the |iHS| statistic may continue to identify regions of high LD surrounding the selected site, but may not detect selection at the selected region itself because fixation will eliminate variation at and near the selected site.

In this study we scan the genome of a diverse set of cattle breeds including dairy and beef breeds based on the 50 K SNP panel. Besides identifying selection footprints common to all breeds, these analyses examine how divergent directions of positive selection may have affected the genomic pattern of those breeds. Our analyses focus primarily on two haplotype and site frequency based statistics: the |iHS| and F_ST _statistics. These tests were chosen because previous power analyses suggest they are largely complementary--|iHS| has good power to detect selective sweeps at moderate frequency, while in contrast, F_ST _is most powerful to detect selection on fixed variation [14]. Applying the |iHS| test with a new Bayesian method of F_ST_, we report a panel of 236 regions putatively subject to recent positive selection confirming the higher differentiation index and longer haplotype consistency for a strong QTL recently detected in Holstein cattle.

## Results

### Marker and LD statistics

Table 1 presents a descriptive summary of data characteristics across breeds for data set I. The average observed heterozygosity and mean MAF were similar in all dairy and dual purpose breeds, while the MAF was generally lower and more variable in beef breeds. The second data set consisted of 40,595 common SNPs typed in 5 breeds which covered 2544.1 Mbp of the genome (Btau 4.0 assembly) with 62.68 ± 58.3 Kbp average adjacent marker spacing. Analysis of the entire panel of across-breed SNPs revealed a non uniform distribution of allele frequencies by breed (results not shown).

**Table 1:** Genome wide summary of marker statistics for the breeds used in LD based analysis (data set I)

Breed	SNP (n)^1^	MAF (%)	ObsHET (%)	Inter-marker distance (kb)	Max gap (kb)
Holstein	39474	28.2 ± 13	37.2 ± 12	64.45 ± 62.5	2081.4
Brown Swiss	35226	27.7 ± 13	36.6 ± 13	72.26 ± 72.8	2081.4
Simmental	37976	27.5 ± 13	37.0 ± 12	67.06 ± 69.8	2145.7
Australian Angus	44938	24.3 ± 15	32.3 ± 16	56.70 ± 52.4	2081.5
Brahman	45173	16.4 ± 14	23.7 ± 17	56.40 ± 51.3	1677.8
Belmond Red	47416	24.1 ± 15	32.3 ± 16	53.74 ± 47.9	1677.8
Hereford	45322	25.5 ± 15	34.1 ± 16	56.22 ± 52.1	2081.5
Murray Gray	41369	24.4 ± 15	33.3 ± 17	61.52 ± 59.0	2081.5
Santa Gertrudis	46809	23.6 ± 15	31.7 ± 17	54.44 ± 48.9	1677.8
Shorthorns	42280	21.7 ± 15	28.5 ± 16	60.26 ± 56.9	2081.5

^1 ^The number of polymorphic SNPs left for final analysis after filtering

**Figure 1:**
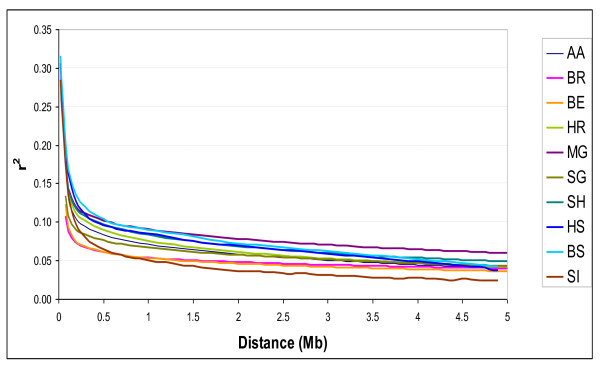
**Decay of LD as a function of inter-marker distance in dairy and beef breeds**.

We compared the extent of LD among breeds. In order to visualize the decay of LD we plotted *r*^2 ^as a function of inter-marker distance (Figure 1). As expected, the level of pair-wise LD as measured by r^2 ^decreases with marker distance within each breed. The decrease is more or less pronounced across the different breeds up to a rather high average value (0.05) at large distances (> 3Mb).

### Signatures of positive selection revealed by |iHS|

**Figure 2:**
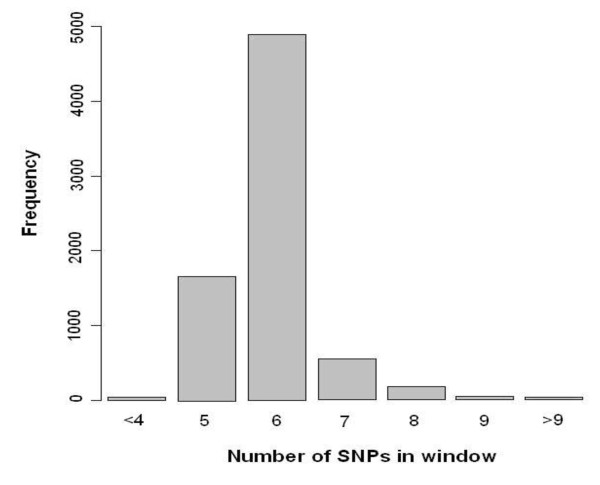
**Distribution of the number of SNPs in 500 kb windows sliding over the genome of breeds in data set I**.

To identify genomic regions that may have been targets of recent selection, we calculated |iHS| for each SNP across the genome of the breeds in the first data set. To facilitate comparisons of genomic regions either within dairy and beef groups or across breeds we split the genome into non-overlapping segments of 500 kb and averaged, in each segment, the |iHS| scores over the SNPs located in each window. 500 kb was chosen as the window size so as to have a sufficient number of SNPs in a window. Figure 2 presents the distribution of the average number of SNPs in windows sliding over the genome of breeds in data set I. We chose this length because of the longer extent of LD in cattle compared to humans, in which the window length used is commonly around 200 Kb [11,12].

We tested 5099 and 5055 sliding windows in beef and dairy groups respectively, involving a total of 49'559 |iHS| values. The mean |iHS| value was 0.74 and the highest estimated value was 3.41 for a region on chromosome 6 in BS. Across breeds, a total of 109 extreme windows exceeded the |iHS| value 1.96 with 19, 27, 9, 10 and 17 outliers in HS, BS, AA, HR and SI, respectively (Additional file 1, Table S1).

**Figure 3:**
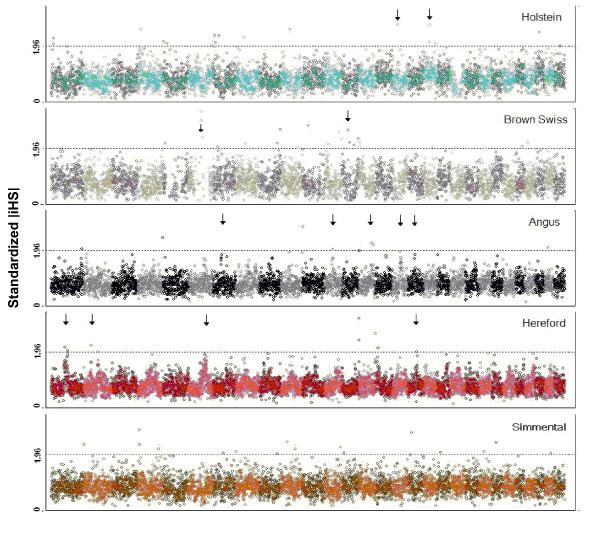
**Genome wide distribution of |iHS| values.** Holstein and Brown Swiss representing dairy vs. Australian Angus and Hereford representing beef breeds and Simmental being a dual purpose breed. Each dot represents a window of 500 Kb and arrows display the clustered signals. Dashed lines are cutting the upper 0.05 of the |iHS| values.”

In order to visualize the chromosomal distribution of outlier signals, we plotted the |iHS| statistic against the genomic position for all breeds (Figure 3 and Additional file 2, Figure S1). A panel of clustered signals representing strong evidence for selective sweeps appeared in a number of breeds. We found evidence of selective sweeps in two regions in HS and two regions in BS. There were also five distinct clusters of |iHS| signals across the genome of AA and four clusters in HR. The clustered signals also overlapped among breeds in some cases (Figure 3, Additional file 2, Figure S1 and Table 2). The regions with clustered signals reflect high values of LD and a slower decay of haplotype homozygosity for a long stretch around the alleles undergoing selection. It is evident that the signals are non-uniformly distributed across chromosomes and chromosome segments.

**Table 2:** Summary statistics for windows representing extreme |iHS| and F_ST_

Chr	Position (Mbp)	|iHS| or *F*_*ST*_^*1*^	Breed^2^	Gene/entry^3 ^(n)	Candidate Gene	Gene name or function
1	79-81.5	2.10	HR	6	SST	Somatostatin
2	34.5-36	2.26	HR	6	GCG	Glucagon
					FAP	Fibroblast activation protein, alpha
2	70-73	2.06	MG/BE/SH/BR	5	-	-
6	61.75-62.75	3.41	BS	13	UGDH	UDP-glucose dehydrogenase
					APBB2	Amyloid beta (A4) precursor protein-binding, family B, member 2 (Fe65-like)
6	80-83		HR	9	SRD5A2L2	Lipid metabolism
7	39-41	1.90	AA	15	COL23A1	Collagen, type XXIII, alpha 1
					MGAT1	Fertilization and early development of the embryos
10	29-31	2.24	BE/SH	8	ACTC1	Actinin, Involved in the formation of filaments
12	36-38	2.03	AA	19	ATP12A	ATPase activity
13	30.5-31.5	2.68	BS	8	TRDMT1	Cysteine and methionine metabolism
14	64-65	2.02	AA	6	MATN2	Developing cartilage rudiments
16	19.75-20.25	2.60	HS	2	SPATA17	Spermatogenesis associated 17
16	39-40	1.98	AA	14	NMNAT1	Methylenetetrahydrofolate reductase (NADPH) activity
17	31-32.5	2.05	AA/HR	15	PGRMC2	Progesterone receptor membrane component 2
18	57.25-57.75	2.20, *0.78*	HS	30	SIGLEC5,8,10	Sialic acid binding Ig-like lectin 5, 8, 10
1	12-13	*0.92*	-	0	-	
2	111.5-112	*0.98*	-	11	ABCB6	ATP-binding cassette, sub-family B (MDR/TAP), member 6
					GLB1L	Galactosidase, beta 1-like
3	119.2-119.7	*0.92*	-	11	SMCP	Sperm mitochondria-associated cysteine-rich protein
7	53.25-53.75	*0.74*	-	4	FGF1	A growth factor which stimulates growth or differentiation, key role in embryonic development
9	42-43	*0.78*	-	12	LACE1	Lactation elevated 1
					PPIL6	Peptidylprolyl isomerase (cyclophilin)-like 6
13	53.5-54	*0.98*	-	7	SIRPA	Signal-regulatory protein
16	4.75-5.25	*098*	-	5	-	-
17	39.5-40.5	*0.98*	-	4	-	-
18	58.25-58.75	*0.98*	-	15	-	-
20	15.25-15.75	*0.92*	-	8	ADAMTS6	-
22	35.25-35.75	*0.77*	-	3	-	-

^1 ^*F*_*ST *_values are in italic

^2 ^Breed name abbreviations are as follows: Australian Angus (AN), Belmond Red (BE), Brahman (BR), Brown Swiss (BS), Hereford (HR), Holstein (HS), Murray Gray (MG), Santa Gertrudis (SG), Shorthorns (SH)

^3 ^Other kind of entries like mRNA, protein, etc submitted in NCBI.

To gain insight into the reliability of our analysis, we compared the |iHS| scores between Angus populations in Australia and Canada and the United States. To this purpose genotypes from 103 North American Angus were used. Because of the smaller sample size and subsequently a larger number of excluded loci (see Material and Methods) only 18'772 SNPs were left for further analyses. Of the total of 12'871 SNPs common between CA and AA, only 107 |iHS| scores overlapped in the 10% upper tail of the empirical distribution, thus basically indicating no major overlap of the regions detected to be under selection.

**Figure 4:**
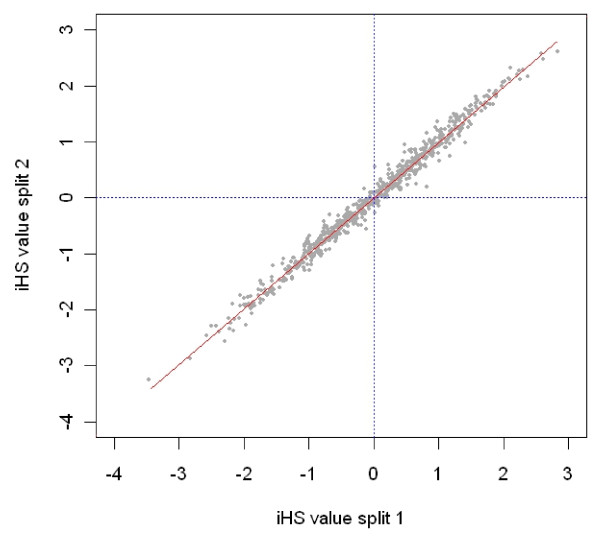
**Cross-validation of |iHS| scores in Holstein data set**. The |iHS| scores from a randomly chosen half data set animals (split 2) are plotted against the other half of the data (split 1).

To assess the background of this result we conducted a cross-validation test [15] regarding the accuracy of |iHS| scores in the Holstein cattle. For this, the Holstein data set was split at random into two data sets, and |iHS| scores calculated from both data sets were found to be in very good agreement (Figure 4). The discordance observed in the two Angus populations could be due to the sparser inter-marker intervals in the North American Angus which may lead to inefficient estimates of |iHS| scores. However, this difference can also be caused by a different genetic composition of the two populations as well as by different selection pressures in the two environments.

### Exploring the differentiated loci

We then investigated evidence for positive selection by assessing variation in allele frequency among populations, using the new Bayesian method proposed by Gianola et al. (2010) [16]. Data set II was used for this purpose. Several comparisons were made, varying the breeds and the sets of SNPs that were included. Summarized pairwise population comparisons of F_ST _values are shown in Table 3. The F_ST _values varied from 0 to 1, which at the extreme represent identity (F_ST _= 0) or fixation of alleles in different populations (F_ST _= 1). The mean posterior distribution of F_ST _values between dairy breeds and between beef breeds respectively, was different from that between dairy and beef breeds. F_ST _between HS and CA was estimated as 0.27 ± 0.01 and between CA and PI as 0.02 ± 0.01. Fixation index estimated between two dairy breeds, HS and BS, was 0.05 ± 0.01.

**Table 3:** Summary statistics of the pair-wise estimates of F_ST _and clustering information

	HS			BS			SI			CA		
	
	F_ST_	K^1^	L^2^	F_ST_	K	L	F_ST_	K	L	F_ST_	K	L
BS	0.05	5	4878									
SI	0.04	4	7796	0.04	5	7691						
CA	0.27	3	12106	0.29	4	5571	0.28	3	10882			
PI	0.27	3	19442	0.28	3	18637	0.27	3	8867	0.02	7	2247

^1 ^Number of clusters

^2 ^Number of SNPs with largest F_ST _values representing the first cluster of loci

In a further step estimates of F_ST _values (in this case, posterior means) per locus were clustered into groups. The expectation was that these clusters might be representative of different processes taking place in the populations such as balancing or directional selection, neutrality or any other specific process. The structure of clustering was explored by fitting a sequence of finite mixture models to the means of posterior distribution of F_ST _values for each locus. Mixture model parameters were estimated by maximum likelihood via the expectation-maximization algorithm in the FlexMix package [17] in the R project. Results of mixture model analysis, by number of clusters favored by the average information criterion (AIC) and the number of loci representing the first cluster (a fraction of loci with largest F_ST _values) in each comparison, are shown in Table 3. In a breed-by-breed comparison of F_ST_, loci were classified into 3 to 7 clusters, possibly reflecting selection footprints left by different evolutionary forces.

**Figure 5:**
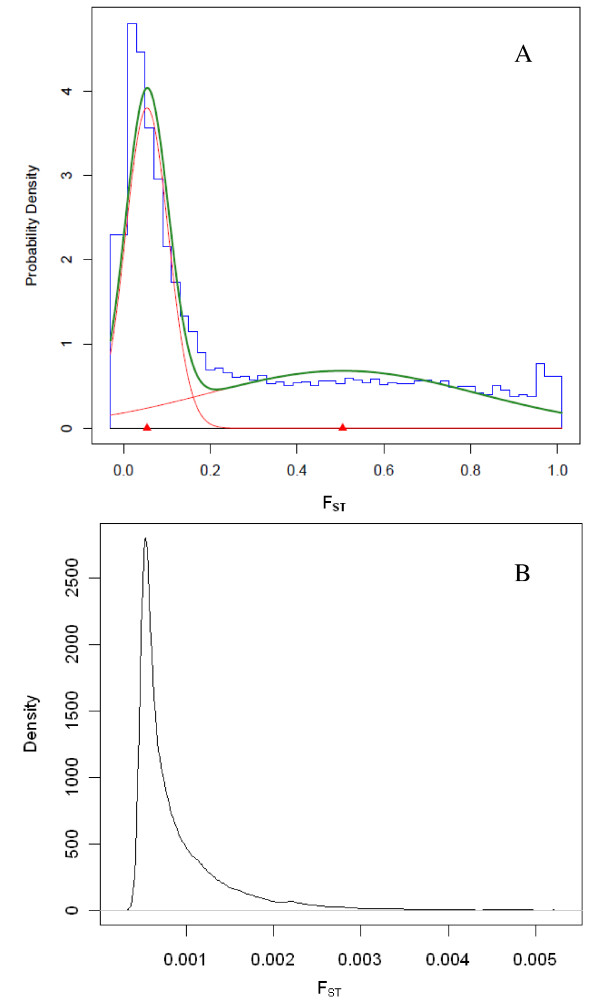
**Density distribution of FST values.** A. Histogram (blue) of the posterior means of F_ST_ values over loci between two dairy (HS and BS) and two beef breeds (CA and PI) and densities of the underlying mixture of two normals (green) and the respective components (red). B. Density plot of 39'474 F_ST_ values between two randomly derived Holstein sub-populations.”

To determine if recent selection was responsible for the differences in allele frequencies between dairy and beef breeds, we examined F_ST _among HS and BS versus CA and PI. In total, 4.3% of the posterior F_ST _means among the 4 populations were < 0.01, 27.1% of the F_ST _values were equal to or greater than 0.5 and the average F_ST _was 0.3. Using Akaike's information criterion as a gauge for model comparison, genome-wide estimates of F_ST _were clustered into two groups, one representing 19'471 putatively neutral loci, and another one included 21'124 loci possibly corresponding to genomic regions affected by selection (Figure 5A).

To address this in some further detail, we partitioned the Holstein population randomly into two sub-populations, then estimated F_ST _and plotted the densities. As shown in Figure 5B, F_ST _values between two sub-populations of no divergence derived from the same breed resulted only in a unimodal distribution indicating a uniform mode of selection over all evaluated loci.

Signatures of selection can be recognized when adjacent SNPs all show high F_ST_, due to the hitch-hiking effect, implying divergent selection between breeds, or where adjacent SNPs all show low F_ST_, implying balancing selection between breeds. Therefore, to facilitate comparisons of genomic regions within or across dairy and beef groups and to reduce locus-to-locus variation in the inference of selection we averaged the F_ST _values into the non-overlapping windows of 500 kb across the genome. Evidence of the positive selection was assumed for windows in the extreme 2.5% of the empirical distribution which resulted in 127 significant windows (Additional file 1, Table S2).

**Figure 6:**
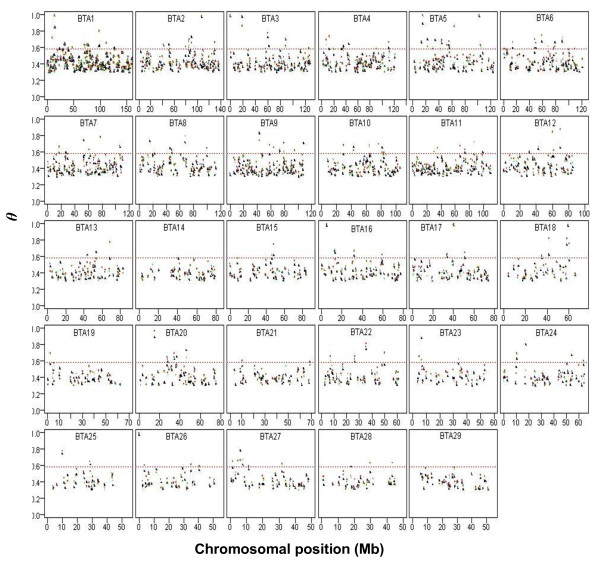
**Genome-wide distribution of F_ST_ signals.** Windows with F_ST_ > 0.3 in all pair-wise comparisons, indicating the genomic position of the most diverse regions between dairy and beef breeds. Blue, black, red, and green dots represent F_ST_ values for HS-CN, HS-PI, BS-CN, and BS-PI, respectively, in each window. Dashed lines display the threshold level of 2.5%.“

To identify differentiated windows between dairy and beef genomic background pairwise F_ST _comparisons denoted as HS-AN, HS-PI, BS-AN and BS-PI were examined and plotted across the genome (Figure. 6). All in all, 29% of the genomic windows with a differentiation index > 0.3 overlapped in the four breed comparisons. Bovine chromosome (BTA) 9 with 80 windows covering 0.35 of the chromosome and BTA25 with 23 windows spanning on 0.26 of the chromosome presented the largest and smallest degree of differentiation in the genome. Figure 6 depicts the genome wide map of F_ST _windows indicating the genomic position of the most diverse regions.

### Genomic annotation

We investigated the genomic regions containing extreme |iHS| and F_ST _values using the fourth draft of bovine genome sequence assembly (Btau 4.0). A subset of genes and ESTs located in each region were identified. We screened this list for the biologically most interesting candidate genes in each region. Table 2 summarizes the statistic estimated as well as the list of genes for 25 genomic regions presenting the most extreme peaks across breeds. Some regions overlapped with genes previously suggested being under selection. For example on chromosome 18 in the Holstein population, an outlier of |iHS| scores was in the interval 57.25-57.75 Mb. This interval contains Sialic acid binding Ig-like lectin 5 and Zinc finger protein 577 genes which recently were reported as candidates to have a strong effect on productive life and fertility traits in Holstein cattle [18].

The window with the largest |iHS| value (3.41) was observed in BS spanning 61.75-62.75 Mb on chromosome 6. Of the 13 genes/ESTs in this region, UGDH (which acts in the carbohydrate metabolism pathways) may be a possible candidate to affect feed efficiency traits. Another strong |iHS| cluster which harbors the Somatostatin (SST) gene was observed on chromosome 1 in HR. Strong evidence of a sweep reflected by a set of windows was observed in the region 80-83 Mb of BTA6 in the vicinity of the SRD5A2 gene. The enzyme steroid 5-alpha-reductase converts testosterone into dihydrotestosterone and a polymorphism in this gene was shown to moderately increase the proportion of progressively motile spermatozoa in normozoospermic men [19]. We also found four clusters of outliers on BTA16 and BTA17, BTA2 and BTA10 which overlapped among some beef breeds.

## Discussion

The high level of observed phenotypic variation among domestic cattle is a result of both neutral demographic processes, weak but sustained natural selection and strong short-term artificial selection for divergent breeding goals. The task of separating these processes and identifying genes under the influence of artificial selection can be challenging. The efforts to identify genes affected by selection have so far been concentrated on species with well-characterized genomes, such as *Drosophila *and humans [11,20]. The cattle genome offers an opportunity to test the power of genome-wide analyses, as it has extensive LD [2,21] caused by intensive selection, and it is expected that selection footprints would be correlated with genes affecting production traits or fitness. However, it must be noticed that extensive LD can also result from other causes, like admixture or genetic drift, both factors being prevalent in farm animal populations and thus making the detection of selection signatures a challenge.

**Figure 7:**
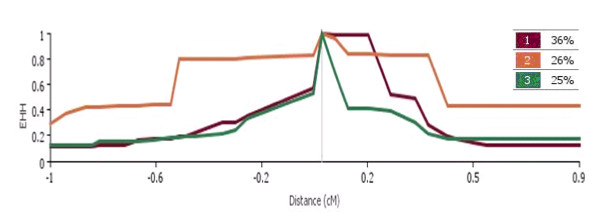
**Frequencies of the haplotypes segregating in the region of extreme |iHS| in the interval 57.25-57.75 Mb on BTA18 in Holstein cattle**. The extent of haplotype homozygosity was estimated by Sweep v.1.1 [11].

In this study we presented an application of two complementary statistics of selection signatures in a diverse set of dairy and beef breeds. In the first step, regions of the genome that contained targets of putative positive selection revealed by long range LD were defined as windows in the extreme of the empirical distribution of the |iHS| statistic. This criterion resulted in 109 significant windows (P ≤ 0.05). These signals generally differ from those reported by the Bovine HapMap consortium [2]. This is probably due to the differences in sample size and marker densities between studies which both could limit accurate estimates of |iHS|. Mapping the corresponding genomic regions to the cattle genome sequence resulted in a large number of adjacent loci. The list of genes with signatures of positive selection was significantly enlarged by those involved in the biological processes such as anatomical structure development, muscle development, metabolism of carbohydrates and lipids, spermatogenesis and fertilization. We refined the complete list for the most important genes in the region of clustered signals that may have functional relevance for economic traits. A notable observation in this study is a strong selection signal confirmed by both |iHS| and F_ST _analyses in the vicinity of Sialic acid binding Ig-like lectin 5 gene on BTA18. This QTL was recently reported to have large effects on calving ease, several conformation traits, longevity, and total merit in Holstein cattle [18]. We observed that other haplotypes present in this region display a shorter extent of homozygosity, indicating abundant historical recombination (Figure 7). Therefore, the long stretch of homozygosity observed in this region presumably is not simply due to a low local recombination rate but presumably reflects the combination of strong and recent selective pressure, pushing the beneficial mutation rapidly towards high frequency with a long conserved haplotype surrounding it. Although the low heritability of most of the aforementioned traits has not made them a primary breeding goal in selection programs, it could be hypothesized that applying sustained but weak negative selection against these traits has increased the frequency of favorable alleles and surrounding haplotypes in the Holstein population.

A cluster of signals reflecting strong evidence of selection was also observed in the vicinity of the Somatostatin gene on BTA1. We also found clusters of outliers which overlapped among some beef breeds (Additional file 1, Table S1). These results show a panel of interesting candidate genes such as SPATA17, MGAT1, PGRMC2 and SRD5A2 in the region of clustered signals which belong to a number of functional categories relevant to reproduction, including gamete generation, embryo development and spermatogenesis, and genes in these categories may be strong candidates for selection for fertility traits. These results generally are consistent with the observations of Flori et al. (2009) [3]. Another interesting observation was the strong evidence for selection in the region of genes related to muscle formation (e.g., ACTC1, COL23A1, MATN2, and FAP) in beef breeds. For example polymorphisms in the genes encoding Actinin are among the best characterized athletic-performance associated variants in human endurance athletes [22,23]. Evidence for positive selection in the genomic region surrounding muscle related genes has also been reported in racing horses [24] and humans [25]. The presence of genes like Actinin, Collagen and Fibroblast activation protein as well as the gene responsible for developing cartilage rudiments in positively selected regions in beef cattle (Table 2) supports the supposition that selection for muscle related phenotypes has played a major role in the shaping the beef cattle. A better understanding of the role these genes play in the development, strength and integrity of muscles may contribute to improved knowledge of musculoskeletal traits and developing new marker systems for beef cattle breeding. Consistency of our observations with previous reports [3,24] may suggest general themes about the types of genes that have been targets of positive selection in cattle.

We also optimized a new Bayesian approach for exploring the level of genetic differentiation to infer the selection signatures against the genome as a whole. This algorithm is able to deal with a large battery of marker information via probabilistic clustering of F_ST _values. After examining F_ST _among HS and BS versus CA and PI breeds using Akaike's information criterion it appears likely that genome-wide estimates of F_ST _are clustered into two groups, one representing putatively neutral loci, and another one (possibly) corresponding to genomic regions affected by selection. Annotation of the genes underlying the regions with extreme F_ST _does not appear to reveal many strong candidates for positive selection with the possible exception of the SMCP and FGF1 genes (Table 2). A receptor of the latter gene (FGFR3) showed evidence of selection in a genome-wide sweep mapping study using F_ST _among dog breeds [26]. This gene is responsible for achondroplasia (shortened limbs) in humans. As an explanation we suggest that selection may work on genes that were not considered the primary targets of selection so far. Some extreme peaks were observed in presumed gene deserts which may reflect selection acting on uncharacterized regulatory regions or simply fixation of non-coding DNA by genetic drift.

We found that 56 of the 127 significant F_ST _values lie in poor gene content regions, defined by the frequency of coding sequences in the bracket of 1 Mb surrounding the F_ST _signal. To test whether this observation is a systematic deviation from the expected, we sampled 127 random positions with matching frequencies on the chromosomes, i.e. since 11 significant F_ST _values were observed on BTA1, we also sampled 11 random positions on that chromosome. Of these 127 random positions, 35 were positioned in regions with poor gene content applying the same definition. The difference was tested with a χ^2 ^test revealing a significant difference on the 5 per cent error level.

This observation is consistent with the studies of Flori et al. (2009) [3], and Gu et al. (2009) [24] which reported F_ST _signals in poor gene content regions in genome wide analyses of cattle and thoroughbred horse, respectively. Thus, these results in combination with the observations from Voight et al. (2006) [12], Carlson et al. (2005) [27] and Wang et al. (2006) [28] on human population data suggest that non-coding regions may have been important for adaptive evolution.

We examined the validity of F_ST _analysis by testing ten candidate major genes in our data set. The results revealed F_ST _values larger than expected (P < 10%) for regions harboring the Casein cluster, GHR, STS, LP, IGF-1 and MSTN genes which are supposed to be targets of artificial selection. The observation of selection evidence in the region of the GHR gene on BTA20 is consistent with the reports of Flori et al. (2009) [3] and Hayes et al. (2009) [4], the latter based on a study comparing Angus and Holstein. The presence of the longer than expected haplotype homozygosity in this region was also observed in Holstein cattle [6]. Two regions on BTA2 and BTA5 in the vicinity of ZRANB3, R3HDM1 and WIF1 genes known to affect feed efficiency and mammalian mesoderm segmentation, respectively [2], also matched with the outlier F_ST _windows in our study.

Overall, the average F_ST _of dairy vs. beef breeds was equal to 0.3 which is substantially higher than the differentiation index reported previously between Holstein and Angus [2,7]. The higher average of F_ST _as well as the similar pair-wise F_ST _within dairy and beef breeds might reflect the dominating influence of a substantial number of fixed SNPs in the pair-wise comparisons of breeds and groups.

The two metrics applied yielded a total of 236 regions putatively subject to positive selection. To investigate how frequently selective events were unique or shared between methods, we assessed the number of overlapping signals. A panel of 6 significant signals was overlapping (Table 4). Interestingly, most of these were found in Holstein cattle, which may reflect a comparatively higher pressure of selective breeding in this breed.

**Table 4:** Overlapping signals revealed by both |iHS| and F_ST _metrics

Chr	Position (Mbp)	Breed	**F**_**ST**_	|iHS|
4	12.5	HS	0.67	2.62
8	40.5	HS	0.59	2.33
10	30.0	SI	0.64	2.48
10	43.5	HS	0.64	2.63
18	58.0	HS	0.78	2.12
22	26.0	BS	0.63	1.99

Overall, comparing our scan for selection with the results of previous genome-wide studies revealed a modest overlap with some notable exceptions. Different hypotheses can be proposed to explain these incongruities. From the methodological point of view, a possible reason could be due to the differences in the computational analyses between the studies. In other words, the statistical tests used in each study are recovering selective events from different time periods and/or for different stages of the selective sweep. Even for tests that should detect similar types of selective events (e.g., scans that identify unusually long haplotypes), low statistical power further decreases the probability of overlap [14]. In addition, most studies report only the most significant results (i.e. outliers in the 1% empirical distribution). Therefore, the results presented in this study are probably a conservative estimate of overlap between studies.

Population demographic history can also impart similar patterns on DNA sequence variation, making inferences on selection difficult. For example, population expansion can lead to an excess of low frequency alleles compared with the number expected under the standard neutral model. Likewise, recent positive selection for a putative mutation may have started from a higher initial frequency of beneficial alleles [29]. Such an allele might e.g. be imported into a breed through crosses with other breeds. In such a case beneficial alleles may be included in diverse haplotypes and LD based estimators would not be able to trace the selection signature. Crossbreeding can also generate false selection signatures, if e.g. a large conserved piece of a chromosome from another breed is mixed with many shorter segments from the original breed.

From the technical point of view, the density of the markers is also critical for the power of such studies and could be a source of discrepancy. It was shown earlier with LD based analyses that core regions are more likely to appear where the marker density is greater than the average [6]. This would imply that the availability of genotyping arrays with an increased genome-wide marker density (by a factor > 10) will allow a more reliable and comprehensive screening of the genome for signatures of selection by LD based tests. Moreover, although sliding window analyses facilitate inferences of selection by reducing locus-to-locus variation, the size of the window is often subjectively determined which can influence the final results and interpretations. One potential refinement would be to adjust window sizes to local levels of LD [30], although it remains unclear how to account for varying levels of LD between populations. Finally, the incongruities can also result from a lack of power given the sample size available for some of the breeds in this study, and complex genomic interactions.

## Conclusions

In this study genomic scans based on site frequency and haplotype data led to the detection of 236 regions putatively subject to recent positive selection in the cattle genome. Our results confirmed the higher differentiation index as well as the longer than expected haplotype consistency in the vicinity of Sialic acid binding Ig-like lectin 5 gene on BTA18, which was recently reported as a strong QTL in the Holstein cattle [18]. However, the overlap between the identified regions via |iHS| with previous studies is modest. Analysis of population differentiation revealed signatures of selection occurring in poor gene content regions, which may reflect selection acting on uncharacterized regulatory regions or simply fixation of non-coding DNA by genetic drift due to the absence of any selection. Besides issues like functionally characterizing suspected targets of selection and identifying causal genes driving signatures of selection observed across large genomic regions, the major challenge remains in developing robust and efficient methods to distinguish true signals from those due to genetic drift. This is especially a challenge for farm animal breeds with small effective population size and thus a considerable impact of genetic drift. One possible solution could be to analyze multiple separate populations with similar breeding goals, hypothesizing that true signal due to selection would overlap across the genomes.

Independent confirmation studies with larger sample sizes and/or higher SNP densities as well as comparisons with other breeds are required as soon as suitable data are available. Further studies should also try to map selection signatures on sex chromosomes, and an attempt should be made to identify gene networks rather than single genes underlying the observed pattern of selection signatures. Our results may be of future interest for identifying signatures of recent positive artificial selection between the cattle breeds or as additional evidence for any polymorphism that shows associations with beef, milk, or functional traits.

## Methods

### Animals

A diverse set of animals collected from Germany, Australia and Canada were used for this study. Table 5 summarizes information of 3876 animals included in our study. The main subset involves Holstein (HS), Simmental (SI) and Brown Swiss (BS) breeds which are part of the total population of cattle genotyped for the genomic selection program in Germany. These breeds are highly selected, essentially for milk production (HF and BS) or for milk and beef (SI). The second subset consisted of 900 individuals collected from 6 beef breeds genotyped in Australia. Another subset of beef cattle included 103 North American Angus (CN) and 43 Piedemontese (PI) collected from Ontario, Canada. The first data set (data set I) consisted of the German breeds mentioned above together with the Australian beef breeds; it was used for LD based analysis in this study. In contrast, the second data set (data set II) included the German breeds together with the Canadian sample and was used for the site frequency approach.

**Table 5:** Description of samples

Breed	Code	Data set		Sample (n)	Country	Purpose
Holstein	HS	I	II	2091	Germany	Dairy
Brown Swiss	BS	I	II	277	Germany	Dairy
Simmental	SI	I	II	462	Germany	Dual-purpose
North American Angus	CA	-	II	103	Canada	Beef
Piedemontese	PI	-	II	43	Canada	Beef
Australian Angus	AA	I	-	232	Australia	Beef
Brahman	BR	I	-	80	Australia	Beef
Belmond Red	BE	I	-	166	Australia	Beef
Hereford	HR	I	-	158	Australia	Beef
Murray Gray	MG	I	-	57	Australia	Beef
Santa Gertrudis	SG	I	-	126	Australia	Beef
Shorthorns	SH	I	-	81	Australia	Beef

### SNP genotypes and data preparation

Semen or blood samples were used as source of genomic DNA. All samples were genotyped using the Illumina Bovine SNP 50 K BeadChip [31]. However, they were genotyped on multiple platforms and at different times. To ensure the highest possible data quality a series of filters were employed to remove lower quality markers and insecure genotypes for individuals. We filtered out samples with ≥ 5% missing genotypes and SNP loci assigned to unpositioned contigs. Genotypes were also discarded if they had quality scores < 95%.

We used only autosomal SNPs with minor allelic frequencies (MAF) ≥ 0.05 in the LD based analysis (data set I). Haplotypes were then reconstructed for each chromosome using default options in fastPHASE [32]. Reconstructed haplotypes were inserted into HAPLOVIEW v4.1 [33] to estimate LD statistics based on pair-wise r^2 ^and construct the blocking pattern in the candidate regions of interest for selection signature analysis. Both paternal and maternal haplotypes were utilized for selection signature analyses.

For the analysis of site frequency spectrum, all SNPs that passed quality control were used in the final analysis, so that loci with MAF < 5% or fixed in some populations were included as well. After quality control and removal of individuals with high proportion of missing genotypes (≥ 5%), data set II consisted of 40,595 common SNPs typed on 2976 animals in 5 breeds (Table 5). The number of heterozygous loci was determined and used to estimate the average heterozygosity for all individuals across the breeds. Allele frequencies and observed and expected heterozygosity for each SNP were also estimated.

### Calculation of |iHS| values

We employed the *iHS *test to evaluate the evidence of positive selection based on haplotype frequencies as described by Voight et al. (2006) [12]. The *iHS *statistic measures the extent of local LD, partitioned into two classes: haplotypes centered upon a SNP that carry the ancestral *versus *the derived allele. For the purpose of this study we used the set of ancestral alleles identified and reported in Matukumalli et al. (2009) [31]. This statistic is applied to individual SNPs and begins by calculating the integrated EHH [11,6], which is defined as the integral of the observed decay of EHH (i.e. the area under the curve of EHH versus distance) away from a specified core allele until EHH reaches 0.05. This integrated EHH (iHH) (summed over both directions away from the core SNP) is denoted *iHH*_*A *_or *iHH*_*D*_, depending on whether it is computed for the ancestral or derived core allele. The unstandardized *iHS *is then calculated as follows:

This quantity is standardized such that it has a mean of 0 and variance of 1 irrespective of allele frequency at the core SNP (see Voight et al. 2006 [12] for details).

Large positive or negative values of *iHS *indicate unusually long haplotypes carrying the ancestral or derived allele, respectively.

### Population differentiation index

In this study we estimated F_ST _= *θ *statistic [9] using a new Bayesian algorithm proposed by Gianola et al. (2010) [16]. The procedure has two steps. First, allelic frequencies are assigned a non-informative prior, leading to less shrinkage of frequencies towards a common value. In maximum likelihood there is no shrinkage at all, an issue criticized by Haldane (1948) [34]. Samples of allelic frequency can be obtained directly because their posterior distributions are tractable analytically and those draws are used to form draws from the posterior distributions of locus-specific F_ST_-parameters, using the parametric definition of F_ST _as a function of allelic frequency (see [16] for more details). This step leads to estimates of the posterior distribution of F_ST _which can be used to explore any underlying structure, presumably caused by different evolutionary forces. In the second step the structure is explored by using features of the posterior distribution of F_ST _(posterior means or transformations thereof) as response variables in a mixed model.

## Abbreviations

iHS: integrated Haplotype Homozygosity Score; EHH: Extended Haplotype Homozygosity; F_ST_: Population fixation index; HS: Holstein, BS: Brown Swiss; SI: Simmental; CA: North American Angus; PI: Piedmontese; AA: Australian Angus; HR: Hereford; SH: Shorthorns; BR: Brahman; BE: Belmond red; MG: Murray Gray; SG: Santa Gertrudis.

## Authors' contributions

SQ carried out the data analyses, drafted and prepared the manuscript for submission. HS supervised the study and contributed in revising and editing the manuscript. BH coordinated in data analysis, provision of data and writing support. DG and FS coordinated in the interpretation of data as well as critically revising the manuscript. GT, SSM and SPM participated in provision of study material and manuscript improvement and also provided administrative support. All authors read and approved the manuscript.

## Supplementary Material

Additional file 1 **Supplementary tables. Table S1**. Genomic regions associated with extreme |iHS| values. |iHS| values averaged over non-overlapping windows of each 500 kb. **Table S2**. Genomic regions associated with extreme F_ST _values (P < 2.5%). F_ST _values averaged over non-overlapping windows of each 500 kb. Click here for file

Additional file 2 **Figure S1**. Distribution of |iHS| values across the genome of beef breeds. Dashed lines display the threshold level of 0.05. Click here for file
