# Supplementary material for: Application of site and haplotype-frequency based approaches for detecting selection signatures in cattle
Source: BMC Genomics. 2011 Jun 16;12:318. doi: 10.1186/1471-2164-12-318 (PMC3146955; doi:10.1186/1471-2164-12-318)
Supplement: Additional file 1 — Supplementary tables. Table S1. Genomic regions associated with extreme |iHS| values. |iHS| values averaged over non-overlapping windows of each 500 kb. Table S2. Genomic regions associated with extreme FST values (P < 2.5%). FST values averaged over non-overlapping windows of each 500 kb. [file 1471-2164-12-318-S1.DOC]

Table S1. Genomic regions associated with extreme |iHS| values. |iHS| values averaged over non-overlapping windows of each 500kb.

| **Position**  **(Mbp)** | **Chr** | **AA** | **BR** | **BE** | **HE** | **MG** | **SG** | **SH** | **HS** | **SI** | **BS** |
| --- | --- | --- | --- | --- | --- | --- | --- | --- | --- | --- | --- |
| 105.5 | 10 | **2.91** | 1.09 | 1.24 | 1.01 | 1.3 | 2.33 | 0.52 | NA | 1.16 | 1.24 |
| 0.25 | 5 | **2.48** | 0.82 | 0.98 | 0.68 | 0.49 | 0.89 | 0.61 | 0.7 | 0.5 | 0.88 |
| 0.25 | 5 | **2.48** | 0.82 | 0.98 | 0.68 | 0.49 | 0.89 | 0.61 | NA | NA | 0.52 |
| 63.5 | 14 | **2.29** | 0.75 | 0.49 | 0.53 | 0.32 | 0.46 | 0.45 | 0.49 | 1.27 | 1.31 |
| 69 | 14 | **2.21** | 0.37 | 0.66 | 0.82 | 0.93 | 1.03 | 0.54 | 0.47 | 0.48 | 0.63 |
| 13.5 | 28 | **2.1** | 0.25 | 0.63 | 0.26 | 0.29 | 1.25 | 0.07 | 0.74 | 0.66 | 0.12 |
| 151 | 1 | **2.06** | 0.51 | 0.45 | 0.99 | 0.41 | 0.92 | 0.75 | 1.43 | 1.24 | 1.09 |
| 38.5 | 12 | **2.03** | 1.17 | 0.77 | 0.44 | 0.53 | 0.6 | 0.24 | NA | NA | NA |
| 84 | 13 | **1.98** | 1.39 | 2.91 | 3.28 | 0.88 | 1.3 | 0.63 | 0.87 | 0.73 | 0.57 |
| 83.5 | 13 | 0.67 | **2.1** | 2.08 | 2.46 | 1.73 | 1.59 | 1.77 | 0.96 | 0.67 | 0.91 |
| 74 | 2 | 0.53 | **2.07** | 1.15 | 0.95 | 0.83 | 0.86 | 1.01 | 0.75 | 0.52 | 0.73 |
| 71 | 2 | 0.99 | **2.05** | 0.73 | 1.5 | 1.67 | 1.6 | 1.73 | 1.01 | 1.04 | 0.37 |
| 12.5 | 18 | 0.6 | **1.98** | 1.21 | 1.5 | 1.17 | 1.01 | 0.7 | 0.85 | 1.05 | 0.55 |
| 51 | 20 | 1 | **1.98** | 0.52 | 0.74 | 0.65 | 1 | 1.14 | NA | 0.39 | 0.80 |
| 84 | 13 | 1.98 | 1.39 | **2.91** | 3.28 | 0.88 | 1.3 | 0.63 | 0.87 | 0.73 | 0.57 |
| 59 | 10 | 0.76 | 1.06 | **2.24** | 1.01 | 0.68 | 0.58 | 0.59 | 0.31 | 0.5 | 0.78 |
| 83 | 13 | 0.55 | 0.9 | **2.2** | 1.25 | 1.45 | 0.97 | 1.28 | 0.65 | 1.19 | 1.26 |
| 69.5 | 5 | 1.05 | 1.12 | **2.03** | 0.9 | 0.99 | 0.58 | 0.78 | 0.78 | 1.35 | NA |
| 39.5 | 25 | 0.58 | 1.07 | **1.99** | 1.47 | 0.69 | 0.42 | 1.14 | 0.52 | 0.65 | 0.61 |
| 138.5 | 1 | 0.96 | 0.77 | **1.98** | 0.56 | 0.75 | 0.48 | 0.54 | 0.56 | 0.55 | 0.07 |
| 30 | 14 | 0.88 | 0.99 | **1.97** | 0.98 | 0.69 | 0.62 | 0.97 | 0.74 | 0.79 | 0.48 |
| 84 | 13 | 1.98 | 1.39 | 2.91 | **3.28** | 0.88 | 1.3 | 0.63 | 0.87 | 0.73 | 0.57 |
| 81.5 | 14 | 1.24 | 0.7 | 1.38 | **2.72** | 1.53 | 0.45 | 1.77 | NA | NA | NA |
| 83.5 | 13 | 0.67 | 2.1 | 2.08 | **2.46** | 1.73 | 1.59 | 1.77 | 0.96 | 0.67 | 0.91 |
| 35 | 2 | 0.67 | 0.85 | 0.59 | **2.26** | 0.98 | 0.53 | 0.6 | 0.65 | 0.7 | 0.61 |
| 68 | 1 | 0.93 | 0.5 | 1.22 | **2.19** | 0.74 | 0.62 | 1.25 | 0.7 | 0.88 | 0.58 |
| 11.5 | 15 | 0.74 | 0.58 | 0.96 | **2.16** | 0.84 | 0.51 | 0.93 | 0.81 | 0.73 | 0.59 |
| 81 | 1 | 0.35 | 0.6 | 1.07 | **2.06** | 0.5 | 1.32 | 0.76 | 0.67 | 1.19 | 0.2 |
| 81.5 | 1 | 0.65 | 0.99 | 1.08 | **2.06** | 0.46 | 1.13 | 0.57 | 0.35 | 1.41 | 0.92 |
| 71.5 | 2 | 0.98 | 0.6 | 0.97 | **2.02** | 1.55 | 0.81 | 1.48 | 0.46 | 0.7 | 0.69 |
| 41.5 | 17 | 0.46 | 0.47 | 0.75 | **2** | 0.86 | 0.98 | 1.4 | 1.09 | 0.85 | 0.03 |
| 54.5 | 14 | 0.6 | 0.31 | 0.84 | 0.57 | **2.29** | 0.71 | 0.47 | 1.19 | 0.89 | NA |
| 17 | 12 | 0.86 | 1.06 | 0.73 | 0.67 | **2.22** | 0.44 | 0.25 | 1.36 | 0.81 | 1 |
| 75 | 2 | 1.18 | 0.19 | 0.64 | 0.61 | **2.14** | 0.99 | 1.55 | 0.62 | 1 | 0.27 |
| 6 | 28 | 0.91 | 0.63 | 1.39 | 0.47 | **2.11** | 0.77 | 1.08 | 0.69 | 0.41 | 0.8 |
| 70.5 | 2 | 0.46 | 0.63 | 0.99 | 1.68 | **2.03** | 0.7 | 1.53 | 0.65 | 0.65 | 0.35 |
| 36 | 16 | 1.49 | 0.48 | 0.78 | 0.69 | **1.98** | 0.65 | 0.7 | 0.40 | 0.40 | NA |
| 105.5 | 10 | 2.91 | 1.09 | 1.24 | 1.01 | 1.3 | **2.33** | 0.52 | NA | 1.16 | 1.24 |
| 17 | 1 | 0.77 | 1.29 | 0.77 | 1.36 | 0.76 | **2.18** | 1.05 | 0.58 | 1.02 | 1.31 |
| 19.5 | 1 | 1.17 | 0.48 | 0.36 | 0.27 | 0.7 | **2.16** | 1.38 | 0.8 | 0.16 | 1.41 |
| 105 | 10 | 1.91 | 1.27 | 0.71 | 1.77 | 1.86 | **2.14** | 1.08 | 0.47 | 0.54 | 0.68 |
| 85.5 | 12 | 0.89 | NA | 0.03 | 1.21 | 1.54 | **2.06** | NA | NA | NA | NA |
| 77 | 14 | 1 | 0.34 | 0.88 | 0.86 | 0.75 | **2.04** | 0.44 | 0.92 | 0.6 | 1.35 |
| 16.5 | 1 | 0.53 | 0.66 | 0.79 | 0.71 | 0.73 | **2** | 1 | 0.72 | 0.77 | 1.15 |
| 35 | 1 | 0.51 | 0.74 | 0.62 | 0.42 | 0.55 | **1.96** | 1.23 | 1.18 | 0.72 | 0.97 |
| 61 | 10 | 0.29 | 0.79 | 1.45 | 0.93 | 1.19 | 0.54 | **2.03** | 0.6 | 0.29 | 0.67 |
| 21 | 16 | 0.23 | 0.53 | 0.62 | 0.61 | 0.39 | 0.17 | 1.03 | **2.83** | 0.8 | NA |
| 30 | 18 | 0.75 | 0.32 | 0.27 | 1.03 | 0.55 | 1.04 | 0.61 | **2.81** | 0.3 | NA |
| 43.5 | 10 | 0.21 | 0.48 | 0.49 | 0.72 | 0.5 | 0.29 | 0.39 | **2.63** | 0.86 | 0.63 |
| 12.5 | 4 | 0.55 | 0.62 | 0.68 | 0.5 | 0.91 | 0.82 | 0.36 | **2.62** | 1.88 | NA |
| 21 | 27 | 0.41 | 0.15 | 0.6 | 1.41 | 0.16 | 0.34 | 0.47 | **2.54** | 0.93 | 0.7 |
| 29.5 | 7 | 0.68 | 0.69 | 0.82 | 1 | 0.75 | 0.9 | 0.86 | **2.41** | 1.6 | 0.44 |
| 9 | 7 | 0.48 | 1.06 | 0.36 | 0.34 | 0.86 | 0.98 | 0.39 | **2.4** | 0.37 | 0.06 |
| 40.5 | 8 | 0.26 | 0.55 | 0.41 | 0.37 | 1.18 | 0.44 | 0.38 | **2.33** | 0.82 | NA |
| 11 | 1 | 0.7 | 0.56 | 0.83 | 0.38 | 0.22 | 0.55 | 0.4 | **2.3** | 0.47 | NA |
| 3 | 5 | 0.42 | 0.42 | 0.39 | 0.78 | 0.32 | 1.56 | 1.01 | **2.17** | 1.25 | 1.28 |
| **Position**  **(Mbp)** | **Chr** | **AA** | **BR** | **BE** | **HE** | **MG** | **SG** | **SH** | **HS** | **SI** | **BS** |
| 25 | 18 | 0.19 | 1.13 | 0.43 | 0.28 | 0.36 | 0.65 | 0.19 | **2.16** | NA | NA |
| 12 | 7 | 0.43 | 0.3 | 0.35 | 0.55 | 0.12 | 0.87 | 0.52 | **2.13** | 0.56 | 0.76 |
| 58 | 18 | 0.62 | 0.82 | 1.01 | 0.84 | 0.87 | 0.63 | 0.56 | **2.12** | 0.05 | NA |
| 21 | 5 | 0.31 | 0.33 | 0.76 | 0.93 | 0.64 | 0.37 | 0.69 | **2.1** | 1.34 | NA |
| 10.5 | 1 | 0.6 | 0.85 | 0.55 | 0.64 | 0.66 | 0.82 | 0.81 | **2.08** | 0.46 | 0.43 |
| 116.5 | 6 | 0.48 | 0.56 | 0.5 | 0.66 | 0.39 | 0.12 | 0.99 | **2.04** | 1.54 | 1.04 |
| 29 | 29 | 0.77 | 0.23 | 0.49 | 0.93 | 0.87 | 0.52 | 0.6 | **2.01** | 0.77 | NA |
| 8 | 7 | 0.45 | 0.55 | 0.47 | 0.47 | 1.72 | 0.67 | 0.34 | **1.99** | 0.75 | 0.89 |
| 79 | 1 | 0.60 | 0.40 | 0.56 | 0.99 | 0.38 | 0.29 | 0.43 | **1.97** | 0.82 | 1.31 |
| 6 | 4 | 0.72 | 0.47 | 0.21 | 0.39 | 0.22 | 0.83 | 0.61 | 1.83 | **2.92** | NA |
| 14 | 17 | 1.19 | 0.33 | 0.62 | 0.54 | 0.73 | 0.73 | 0.47 | 1.05 | **2.83** | NA |
| 30 | 10 | 0.7 | 0.4 | 0.48 | 0.59 | 0.78 | 0.34 | 0.98 | 0.56 | **2.48** | NA |
| 21.5 | 23 | 0.73 | 0.64 | 0.4 | 0.43 | 0.61 | 0.56 | 0.65 | 0.6 | **2.45** | 1.84 |
| 6.5 | 4 | 0.54 | 0.34 | 0.2 | 0.46 | 0.12 | 1.03 | 0.48 | 1.94 | **2.38** | 0.94 |
| 2.5 | 2 | 0.33 | 0.25 | 0.97 | 0.29 | 0.37 | 0.85 | 0.03 | 0.14 | **2.37** | 0.17 |
| 78 | 12 | 0.45 | 0.85 | 0.87 | NA | NA | 0.42 | 1.07 | 1.48 | **2.27** | 1.36 |
| 101 | 4 | 0.83 | 0.38 | 1.09 | 0.4 | 0.68 | 0.18 | 1.32 | 1.26 | **2.21** | NA |
| 67 | 10 | 0.58 | 0.34 | 0.51 | 0.72 | 1.14 | 0.32 | 0.78 | 0.2 | **2.19** | NA |
| 23.5 | 22 | 0.84 | 0.19 | 0.47 | 0.46 | 0.43 | 0.51 | 0.42 | 0.27 | **2.11** | 0.1 |
| 2 | 8 | 0.61 | 0.09 | 0.36 | 0.48 | 0.41 | 0.63 | 0.29 | 1.57 | **2.1** | 1.16 |
| 16.5 | 19 | 0.85 | 0.49 | 0.5 | 0.84 | 0.44 | 0.54 | 0.65 | 0.8 | **2.08** | 1.53 |
| 10.5 | 13 | 1.02 | 0.34 | 0.44 | 0.54 | 0.79 | 0.57 | 0.37 | 1.62 | **2.05** | NA |
| 28 | 28 | 0.69 | 0.84 | 0.65 | 1.3 | 0.84 | 0.45 | 0.37 | 1.56 | **2.04** | 1.33 |
| 48.5 | 7 | 0.38 | 0.67 | 0.87 | 0.59 | 0.6 | 0.38 | 0.19 | NA | **2.04** | NA |
| 87 | 9 | 0.34 | 1 | 1.44 | 0.49 | 1.2 | 0.84 | 0.59 | 0.66 | **2.02** | 0.44 |
| 9 | 17 | 0.99 | 0.86 | 0.68 | 0.83 | 0.90 | 0.64 | 0.56 | 0.34 | **1.96** | 0.74 |
| 62 | 6 | 0.77 | 0.4 | 0.63 | 1.27 | 1.23 | 0.9 | 0.95 | 0.57 | 0.56 | **3.41** |
| 62.5 | 6 | 0.58 | 0.65 | 1.02 | 1.12 | 1.52 | 0.79 | 1.87 | 0.44 | 0.46 | **3.05** |
| 29.5 | 11 | 0.66 | 0.94 | 0.65 | 0.78 | 0.65 | 0.8 | 1.05 | 0.73 | 0.86 | **2.86** |
| 103 | 9 | 0.79 | 0.38 | 0.45 | 0.69 | 0.52 | 0.48 | 0.59 | 1.22 | 0.8 | **2.71** |
| 30.5 | 13 | 0.87 | 0.41 | 0.5 | 0.7 | 0.9 | 0.67 | 0.69 | 0.36 | 1.33 | **2.68** |
| 70.5 | 12 | 0.65 | 0.3 | 0.73 | 0.88 | 0.96 | 1.06 | 0.7 | 0.58 | 0.78 | **2.62** |
| 71.5 | 6 | 1.09 | 0.98 | 0.78 | 1.28 | 1.26 | 0.95 | 0.28 | 0.51 | NA | **2.43** |
| 79 | 13 | 0.62 | 1.04 | 0.97 | 0.8 | 0.31 | 0.46 | 0.45 | 1.17 | 1.17 | **2.36** |
| 79.5 | 12 | 0.72 | 0.65 | 0.63 | 0.85 | 0.51 | 0.55 | 1.04 | 1.05 | 1.27 | **2.33** |
| 0.5 | 14 | 0.39 | 0.76 | 0.75 | 0.21 | 0.26 | 0.26 | 0.54 | 1.1 | 0.75 | **2.22** |
| 36.5 | 13 | 0.55 | 0.78 | 0.61 | 0.52 | 0.67 | 0.42 | 1.02 | 1.31 | 1.58 | **2.22** |
| 10 | 5 | 1.72 | 0.45 | 0.26 | 0.7 | 0.44 | 0.27 | 0.67 | 0.59 | 1.83 | **2.2** |
| 86.5 | 9 | 0.46 | 0.59 | 1.08 | 0.48 | 0.96 | 0.53 | 0.59 | 0.49 | 0.56 | **2.2** |
| 14.5 | 26 | 0.77 | 0.73 | 0.92 | 0.71 | 0.46 | 0.81 | 0.39 | 0.58 | 1.04 | **2.18** |
| 45.5 | 8 | 0.55 | 0.7 | 1.1 | 0.98 | 1.19 | 0.98 | 0.63 | 0.79 | 1.84 | **2.17** |
| 16 | 12 | 0.47 | 0.73 | 0.63 | 1 | 1.23 | 0.85 | 0.74 | 1.26 | 1.31 | **2.13** |
| 57.5 | 13 | 0.53 | 0.47 | 1.13 | 0.62 | 0.73 | 1.3 | 0.23 | 0.74 | 0.73 | **2.12** |
| 30.5 | 25 | 0.97 | 0.47 | 0.6 | 0.77 | 1.15 | 1.13 | 0.71 | 1.14 | 0.87 | **2.07** |
| 2 | 14 | 0.59 | 0.88 | 0.58 | 0.67 | 0.64 | 0.4 | 0.57 | 0.68 | 1.57 | **2.06** |
| 39 | 18 | 0.91 | 0.66 | 0.49 | 0.8 | 0.39 | 0.97 | 0.58 | 1.55 | 0.99 | **2.05** |
| 23 | 7 | 0.35 | 0.58 | 0.5 | 0.31 | 0.38 | 0.68 | 0.59 | 0.65 | 0.98 | **2.03** |
| 24.5 | 20 | 0.82 | 0.9 | 1.13 | 0.91 | 0.83 | 0.4 | 0.62 | 0.7 | 1.05 | **2.02** |
| 85 | 10 | 0.31 | 0.87 | 0.37 | 0.49 | 0.44 | 0.41 | 1.08 | 0.85 | 0.93 | **2.01** |
| 26 | 22 | 0.79 | 0.28 | 0.53 | 0.44 | 0.94 | 0.61 | 0.43 | 0.3 | 0.56 | **1.99** |
| 50 | 26 | 0.96 | 0.98 | 0.71 | 1.39 | 0.85 | 0.69 | 0.58 | 1 | 1.03 | **1.97** |
| 104 | 2 | 0.32 | 1.61 | 0.32 | 0.25 | 0.51 | 0.43 | 0.15 | 0.84 | 0.81 | **1.96** |
| 51.5 | 1 | 0.62 | 0.90 | 1.32 | 0.48 | 0.64 | 0.56 | 0.55 | 0.31 | 1.03 | **1.96** |

Table S2. Genomic regions associated with extreme *θ* values (P < 2.5%). *θ*s averaged over non-overlapping windows of each 500kb.

| **Chr** | **Position (Mb)** | ***θ*** |
| --- | --- | --- |
| 1 | 13.5 | 0.92 |
| 1 | 98 | 0.72 |
| 1 | 47 | 0.63 |
| 1 | 75.5 | 0.63 |
| 1 | 19 | 0.62 |
| 1 | 9 | 0.62 |
| 1 | 111.5 | 0.6 |
| 1 | 32 | 0.6 |
| 1 | 77 | 0.6 |
| 1 | 70.5 | 0.59 |
| 1 | 39.5 | 0.58 |
| 2 | 107 | 0.98 |
| 2 | 90 | 0.68 |
| 2 | 85.5 | 0.65 |
| 2 | 42.5 | 0.64 |
| 2 | 130 | 0.63 |
| 2 | 51 | 0.63 |
| 3 | 19 | 0.92 |
| 3 | 60.5 | 0.76 |
| 3 | 88.5 | 0.66 |
| 3 | 6 | 0.61 |
| 3 | 62 | 0.59 |
| 4 | 12.5 | 0.67 |
| 4 | 111.5 | 0.64 |
| 4 | 36 | 0.63 |
| 4 | 8 | 0.59 |
| 4 | 45.5 | 0.57 |
| 5 | 102.5 | 0.99 |
| 5 | 16 | 0.94 |
| 5 | 53 | 0.68 |
| 5 | 44.5 | 0.68 |
| 5 | 22.5 | 0.67 |
| 5 | 14 | 0.67 |
| 5 | 64 | 0.66 |
| 5 | 56 | 0.65 |
| 5 | 34.5 | 0.61 |
| 5 | 104.5 | 0.58 |
| 6 | 90 | 0.74 |
| 6 | 58.5 | 0.72 |
| 6 | 79 | 0.64 |
| 6 | 14.5 | 0.64 |
| 6 | 47.5 | 0.63 |
| 6 | 50 | 0.63 |
| 6 | 68.5 | 0.6 |
| 6 | 5 | 0.58 |
| 7 | 53.5 | 0.74 |
| 7 | 17 | 0.65 |
| 7 | 106.5 | 0.62 |
| 7 | 77.5 | 0.62 |
| 7 | 61.5 | 0.61 |
| 7 | 27 | 0.6 |
| 8 | 15.5 | 0.73 |
| 8 | 68 | 0.7 |
| 8 | 48.5 | 0.64 |
| 8 | 100 | 0.63 |
| **Chr** | **Position (Mb)** | ***θ*** |
| 8 | 45.5 | 0.59 |
| 8 | 61 | 0.57 |
| 9 | 43 | 0.79 |
| 9 | 58 | 0.63 |
| 9 | 73.5 | 0.62 |
| 9 | 79 | 0.6 |
| 10 | 43.5 | 0.64 |
| 10 | 29.5 | 0.64 |
| 10 | 64.5 | 0.63 |
| 10 | 81.5 | 0.62 |
| 11 | 87 | 0.67 |
| 11 | 67.5 | 0.65 |
| 11 | 74 | 0.62 |
| 11 | 35 | 0.61 |
| 12 | 69.5 | 0.77 |
| 12 | 60 | 0.74 |
| 12 | 43 | 0.66 |
| 12 | 32 | 0.62 |
| 12 | 61.5 | 0.6 |
| 12 | 36 | 0.58 |
| 13 | 54 | 0.99 |
| 13 | 69 | 0.69 |
| 13 | 44 | 0.59 |
| 14 | 57 | 0.57 |
| 15 | 48.5 | 0.68 |
| 15 | 46.5 | 0.6 |
| 16 | 5 | 0.98 |
| 16 | 13 | 0.67 |
| 16 | 32 | 0.65 |
| 16 | 13.5 | 0.61 |
| 16 | 59.5 | 0.6 |
| 17 | 40.5 | 0.99 |
| 17 | 40 | 0.78 |
| 17 | 34 | 0.64 |
| 17 | 51.5 | 0.62 |
| 18 | 59.5 | 0.98 |
| 18 | 57.5 | 0.78 |
| 18 | 42 | 0.73 |
| 18 | 60.5 | 0.66 |
| 18 | 35.5 | 0.62 |
| 19 | 2.5 | 0.64 |
| 20 | 15.5 | 0.93 |
| 20 | 46.5 | 0.69 |
| 20 | 34.5 | 0.66 |
| 20 | 27.5 | 0.62 |
| 20 | 37 | 0.62 |
| 21 | 10.5 | 0.61 |
| 21 | 68.5 | 0.58 |
| 22 | 35.5 | 0.78 |
| 22 | 50 | 0.68 |
| 22 | 26.5 | 0.63 |
| 22 | 7 | 0.59 |
| 23 | 34 | 0.61 |
| 24 | 54.5 | 0.68 |
| 24 | 10.5 | 0.66 |
| 24 | 18 | 0.66 |
| 24 | 64 | 0.59 |
| 24 | 50.5 | 0.57 |
| 25 | 10 | 0.76 |
| 25 | 29 | 0.61 |
| **Chr** | **Position (Mb)** | ***θ*** |
| 26 | 1.5 | 0.62 |
| 26 | 35 | 0.6 |
| 26 | 40.5 | 0.58 |
| 26 | 3.5 | 0.57 |
| 27 | 6.5 | 0.72 |
| 27 | 8 | 0.64 |
| 27 | 11.5 | 0.59 |
| 27 | 32 | 0.58 |
| 27 | 5 | 0.58 |
| 28 | 30 | 0.59 |
| 28 | 43.5 | 0.58 |
