# Supplementary material for: Application of site and haplotype-frequency based approaches for detecting selection signatures in cattle
Source: BMC Genomics. 2011 Jun 16;12:318. doi: 10.1186/1471-2164-12-318 (PMC3146955; doi:10.1186/1471-2164-12-318)
Supplement: Additional file 2 — Figure S1. Distribution of |iHS| values across the genome of beef breeds. Dashed lines display the threshold level of 0.05. [file 1471-2164-12-318-S2.DOC]

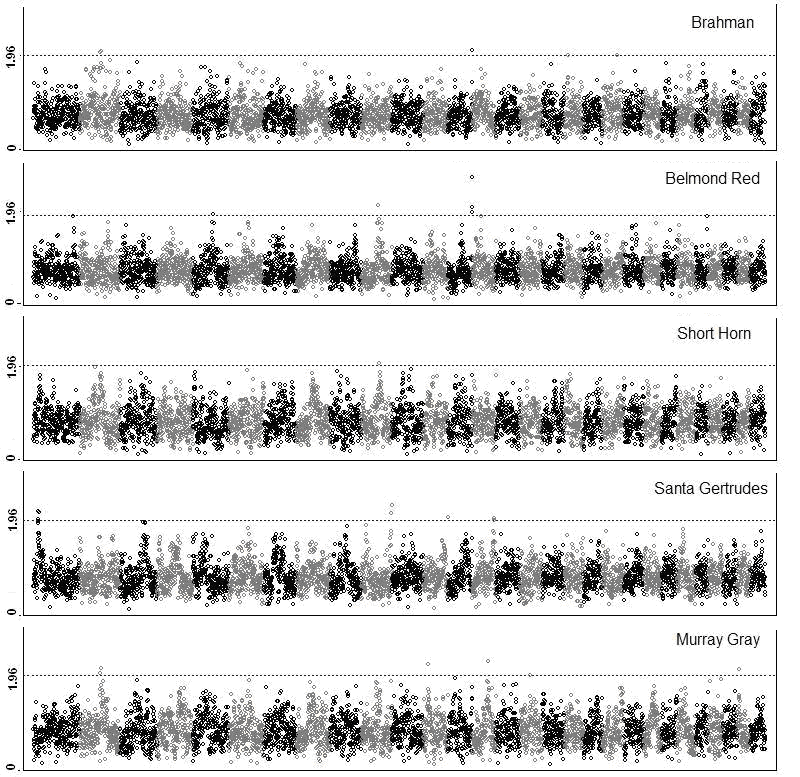


**Standardized |iHS|**

**Distribution of** |iHS| values across the genome of beef breeds. **Dashed lines display the threshold level of 0.05.**
